# Single-Cell Analysis Uncovers a Vast Diversity in Intracellular Viral Defective Interfering RNA Content Affecting the Large Cell-to-Cell Heterogeneity in Influenza A Virus Replication

**DOI:** 10.3390/v12010071

**Published:** 2020-01-07

**Authors:** Sascha Young Kupke, Lam-Ha Ly, Stefan Thomas Börno, Alexander Ruff, Bernd Timmermann, Martin Vingron, Stefan Haas, Udo Reichl

**Affiliations:** 1 Department of Bioprocess Engineering, Max Planck Institute for Dynamics of Complex Technical Systems, 39106 Magdeburg, Germany; ruff@mpi-magdeburg.mpg.de (A.R.); ureichl@mpi-magdeburg.mpg.de (U.R.); 2 Department of Computational Molecular Biology, Max Planck Institute for Molecular Genetics, 14195 Berlin, Germany; vingron@molgen.mpg.de (M.V.); haas@molgen.mpg.de (S.H.); 3 Sequencing Core Facility, Max Planck Institute for Molecular Genetics, 14195 Berlin, Germany; timmerma@molgen.mpg.de; 4 Bioprocess Engineering, Otto von Guericke University Magdeburg, 39106 Magdeburg, Germany

**Keywords:** single-cell analysis, influenza A virus, cell-to-cell heterogeneity, defective interfering particles, single-cell RNA sequencing, next-generation sequencing

## Abstract

Virus replication displays a large cell-to-cell heterogeneity; yet, not all sources of this variability are known. Here, we study the effect of defective interfering (DI) particle (DIP) co-infection on cell-to-cell variability in influenza A virus (IAV) replication. DIPs contain a large internal deletion in one of their eight viral RNAs (vRNA) and are, thus, defective in virus replication. Moreover, they interfere with virus replication. Using single-cell isolation and reverse transcription polymerase chain reaction, we uncovered a large between-cell heterogeneity in the DI vRNA content of infected cells, which was confirmed for DI mRNAs by single-cell RNA sequencing. A high load of intracellular DI vRNAs and DI mRNAs was found in low-productive cells, indicating their contribution to the large cell-to-cell variability in virus release. Furthermore, we show that the magnitude of host cell mRNA expression (some factors may inhibit virus replication), but not the ribosome content, may further affect the strength of single-cell virus replication. Finally, we show that the load of viral mRNAs (facilitating viral protein production) and the DI mRNA content are, independently from one another, connected with single-cell virus production. Together, these insights advance single-cell virology research toward the elucidation of the complex multi-parametric origin of the large cell-to-cell heterogeneity in virus infections.

## 1. Introduction

Influenza A viruses (IAVs) cause respiratory disease and are important human pathogens that can lead to a high morbidity. IAV epidemics occur annually, but occasionally severe pandemics can also arise. IAVs harbor a segmented and single-stranded RNA genome of negative polarity comprising eight individual viral RNAs (vRNAs) [1]. The vRNAs occur encapsidated with multiple copies of the viral nucleoprotein and the tripartite viral polymerase complex [2,3], forming so-called viral ribonucleoprotein (vRNP) complexes [4,5]. Upon infection and import into the cell nucleus, vRNPs are able to catalyze the transcription of viral messenger RNAs (mRNAs) as well as the synthesis of complementary RNAs, which are themselves encapsidated in complementary ribonucleoproteins that serve as a template for the replication of progeny vRNAs [6,7].

Occasionally, large internal deletions or point mutations [8] occur during the replication of the vRNA genome, which give rise to defective interfering (DI) vRNAs. The conventional deletions in DI vRNAs are believed to arise by erroneous translocation of the viral polymerase, called the “copy-choice” mechanism [9,10]. Deletions can comprise hundreds up to roughly two thousand, base pairs. However, DI vRNAs typically retain the terminal 3′ and 5′ ends, which harbor the promotor (important for transcription and replication) and the genome packaging signal [11,12]. Furthermore, deletions in segment 1 (S1)-S3, which encode for viral polymerase proteins, are most frequently observed [11,12,13]. Interestingly, such DI vRNAs are preferentially synthesized and packaged into progeny virions over their cognate full-length (FL) vRNAs, which results in the release of DI particles (DIPs) [14].

Due to the lack of genomic information, DIPs are defective in virus replication and cannot replicate in the context of an infection [11,15]. However, in the case of a co-infection with an infectious standard virus (STV), this defect can be overcome. However, as a result, STV replication is then suppressed, and mainly non-infectious DIPs are produced. This interference is believed to be caused by a replication advantage of the DI genome, which may out-compete the FL vRNA for limited viral or cellular resources [9,10,16]. Furthermore, DI vRNAs are suggested to multiply faster due to their reduced length [11,16,17]. Finally, DI genomes were found to competitively inhibit the packaging of their predecessor FL vRNAs into progeny virions [18,19]. Due to their ability to inhibit STV replication, previous research suggests that DIPs may be used as an antiviral agent [15,20,21,22,23]. Moreover, DIPs can reduce virus yields in cell culture-based vaccine production [24,25], and may affect the efficacy of live-attenuated vaccines [26,27,28].

At the single cell level, virus replication is highly heterogeneous and shows a vast cell-to-cell variability, with up to roughly 1000-fold differences in intracellular viral RNA levels and progeny virus yields [29,30,31,32]. Potential sources that have been discussed to affect or generate such a large heterogeneity are the cell size [29,30,33,34,35], the cell cycle stage [29,35], the innate immune response [36,37,38,39], the between-virus genetic heterogeneity of the infecting virus population [29,40], and the inherent stochasticity of biochemical reactions [31,32,41,42]. Moreover, whole-transcriptome analysis via single-cell RNA sequencing (scRNA-seq) revealed that, for instance, proteins involved in the transcriptional regulation, endoplasmic reticulum (ER) translocation, signal peptide processing and membrane trafficking, ubiquitination, oxidative and ER stress response, and mitochondrially encoded genes can further affect the strength of virus replication in a single cell [37,39,40,43,44]. However, not all causes of the large cell-to-cell heterogeneity in virus infections have been identified, and a detailed comprehension of their complex dependencies remains largely elusive.

DIPs exert strong inhibitory effects on virus replication. Moreover, for a given concentration of DIPs in the infecting virus population, a large variation in the number of infecting DIPs per cell must be assumed, and a certain fraction of individual cells might not even become infected by a DIP. Most likely, this heterogeneity has a significant impact on cell-to-cell variability in progeny virus production.

In this study, we performed single-cell analysis of IAV replication and compared extracellular progeny virus titers and intracellular DI vRNAs, or their derivatives, DI mRNAs, via conventional RT-PCR or scRNA-seq, respectively. Our experiments revealed an extreme cell-to-cell heterogeneity in the content of DI vRNAs and DI mRNAs in IAV infection. In addition, results obtained from scRNA-seq indicate the de novo generation of a diversity of DI vRNAs, observed at the single cell level, which further adds to the large cell-to-cell variability in IAV replication. Moreover, an increased intracellular level of DI vRNAs and DI mRNAs correlated with a low progeny virus titer, and vice versa, which implicates that both deleted RNA species can contribute to the large cell-to-cell heterogeneity in IAV replication. However, we also found some exceptions from this observation, suggesting the presence of additional factors that can affect or generate single-cell diversity in virus release. Finally, we investigated the impact of some of these factors on the cell-specific virus titer. These results may help to unravel the complexity of between-cell variability in virus infections.

## 2. Materials and Methods

### 2.1. Cells and Viruses

Adherent Madin–Darby canine kidney (MDCK) cells (European Collection of Authenticated Cell Cultures, #84121903) were cultivated in Glasgow Minimum Essential Medium (GMEM), supplemented with 10% fetal calf serum (FCS) and 2 g/L peptone. The infection medium comprised GMEM, 2 g/L peptone, and porcine trypsin (5 BAEE U/mL), but no FCS. Cultivations and infections were conducted at 37 °C and 5% CO_2_. Influenza virus strain PR8 was provided by the Robert Koch Institute (Berlin, Germany, #3138). Seed virus titer was determined by 50% tissue-culture-infective dose (TCID_50_) assay on MDCK cells [45], and multiplicity of infections (MOIs) were calculated based on this titer. We used one seed virus for all independent infection experiments.

### 2.2. Isolation of Infected Single Cells

Single cell isolation was conducted as described previously [8,32]. Briefly, confluent MDCK cells (in 9.6 cm^2^ dishes) were infected at an MOI of 10 in 250 µL of infection media. Cells were incubated for 1 h, and dishes were rocked every 20 min to distribute the inoculum evenly. Afterward, we increased the medium volume to 2 mL, and cells were further incubated for 1.5 h. Cells were washed twice with phosphate buffered saline (PBS), then the cells were quickly trypsinized for 10–15 min. The reaction was stopped using cell cultivation media (containing FCS), cells were homogenized, and diluted in infection medium. The diluted cell suspension was added to a 384-well plate (Greiner, Kremsmünster, Austria, #781901) to yield a calculated number of one cell per well (in 50 µL of medium). Incubation was conducted until 12 hpi. Cells were then briefly centrifuged at 150× *g*, and single cells in individual wells were identified by phase-contrast microscopy. Single-cell supernatants were then subjected to plaque assays for quantification of the virus titers. Remaining single cells in the wells were washed twice with PBS. We then added 5 µL of lysis buffer, consisting of a bovine serum albumin solution (Thermo Scientific, Waltham, MA, USA, #B14), diluted to 1 mg/mL, and 1 U/µL RiboLockRNase Inhibitor (Thermo Scientific). The plate was then sealed and stored at −80 °C until single-cell RT-PCR or scRNA-seq.

### 2.3. Plaque Assay

Complete single-cell-derived supernatants were subjected to the plaque assay to investigate the virus titer (PFU/cell). Specifically, we used two dilutions that comprised 90% and 10% of the sample. Adherent MDCK cells in 6-well plates were incubated with 250 µL of each dilution for 1 h. Plates were rocked every 20 min to distribute the inoculum evenly. We then removed the inoculum, and well cells were overlaid with 1% of agar in an infection medium. After four days, cells were fixed with methanol, and stained with a 0.2% crystal violet solution. Plaques were then counted using standard light microscopy.

### 2.4. Single-Cell reverse transcription polymerase chain reaction (RT-PCR)

Whole single-cell lysates (5 µL) were used for RT-PCR to investigate the presence of intracellular DI vRNAs. Specifically, we examined S1–S3, as DI vRNAs are typically predominantly generated on these segments. The procedure was based on a previously published method [24], which was further optimized to achieve single-cell sensitivity. For RT, a universal primer Uni12 (Table 1) was used [46], which can bind to the conserved 3′ end of every IAV vRNA, which facilitates the synthesis of all eight cDNAs in one reaction. For PCR, individual reactions for each segment were carried out. For this, the primers (Table 1) encompass the 3′ or 5′ ends in conjunction with a segment-specific part of the vRNA, which enables the specific amplification of the complete vRNA segments.

For RT, 5 µL of the sample was mixed with 0.5 μL dNTPs (10 mM) and 0.5 μL primer (10 mM), and filled up with nuclease-free water to 7.25 μL. The reaction mix was incubated at 65 °C for 5 min and then 4 °C for 5 min. Afterward, we added 2 μL of 5× Reaction Buffer, 25 U (equal to 0.25 µL) RevertAid H Minus Reverse Transcriptase, 10 U (equal to 0.25 µL) RiboLock RNase Inhibitor, and 0.25 μL nuclease-free water (all reagents from Thermo Scientific). RT reaction was performed at 42 °C for 60 min, and terminated at 70 °C for 10 min. Samples were either stored at −20 °C, or immediately subjected to PCR.

For PCR, 3 μL of the cDNA sample was mixed with 6 μL of 5× Phusion GC Buffer, 3 μL MgCL_2_ (10 mM), 1.5 μL dNTPs (10 mM), 1.5 μL of each primer (10 μM), 0.6 U (equal to 0.3 μL) Phusion Hot Start II DNA Polymerase, and 13.2 μL nuclease-free water (all reagents from Thermo Scientific). PCR was initiated at 98 °C for 3 min, followed by 40 PCR cycles at 98 °C for 25 s, 58.5 °C for 45 s, and 72 °C for 2 min. Final elongation was performed at 72 °C for 10 min. Subsequently, agarose gel electrophoresis was used to visualize the PCR products.

For quantification of the results from RT-PCR and subsequent agarose gel electrophoresis, the image processing program ImageJ (National Institutes of Health, Bethesda, MD, USA) was used. More specifically, the software was used to quantify the signal intensities of the agarose gel images of the signals corresponding to the DI and FL vRNAs. For this, we (i) subtracted the background of the gel image, (ii) selected and transformed each band to a graph that displayed the grey value against the distance of the gel, and (iii) determined the area under the curve (AUC) of each band. Subsequently, all extracted AUCs of each band were normalized against the AUC obtained from a specific band (300 bp) of the DNA ladder. This was necessary for comparability of the numeric values obtained across different agarose gels. Subsequently, we calculated the quantity ratio of DI to FL vRNAs for each single cell by dividing the sum of normalized AUCs corresponding to DI vRNAs on S1–S3 by the sum of normalized AUCs belonging to the FL vRNAs on S1–S3.

### 2.5. Quantification of Ribosomes

The ribosome content of infected single cells was quantified using real-time RT-qPCR. The quantification was based on the detection of 18S rRNA, which is associated with the small ribosomal subunit at exactly one 18S rRNA molecule per ribosome [47].

For RT, 1 µL of the single-cell lysate was combined with 0.5 µL of dNTPs (10 mM) and 0.5 µL of a random hexamer primer (100 µM), and filled up to 6.5 µL with nuclease-free water. Incubation was performed at 65 °C for 5 min and then 4 °C for 5 min. We then added 2 μL of 5x RT Buffer, 10 U (equal to 0.25 µL) RiboLock RNase Inhibitor, 25 U (equal to 0.25 µL) “Maxima H Minus Reverse Transcriptase”, and 1 μL nuclease-free water (all reagents from Thermo Scientific). RT reaction was performed at 25 °C for 10 min and 50 °C for 30 min. Termination of the RT was conducted at 85 °C for 5 min. cDNA samples were either stored at −20 °C, or immediately subjected to qPCR.

For qPCR, the Rotor-Gene Q real-time PCR cycler (Qiagen) was used and primers specific for 18S rRNA: “18S for” (5′-CGGACAGGATTGACAGATTG-3′) and “18S rev” (5′-CAAATCGCTCCACCAACTAA-3′). For this, we mixed 5 µL of the 2x Rotor-Gene SYBR Green PCR Kit (Qiagen), 0.5 µL of each primer (10 µM), 1 µL of nuclease-free water, and 3 µL of cDNA sample. The following temperature profile was used for qPCR: initial denaturation at 95 °C for 5 min, followed by 40 PCR cycles of (i) 95 °C for 10 s and (ii) 62 °C for 20 s. Final denaturation was performed at 95 °C for 15 s. Subsequent melting curve analysis was conducted at temperatures between 65 to 90 °C.

For the assessment of the single-cell expression of 18S rRNA, the fold change of a single cell’s expression over the average 18S rRNA single-cell expression was calculated. For this, we used the C_T_ values from single-cell measurements ($C_{T\_SC}$) and the arithmetic mean of the C_T_ value of all single-cell measurements ($C_{T\_\bar{x}}$) within a measurement run.

(1) $$Fold\;change\;over\;average=\;2^{-\left(C_{T\_SC}\;-\;C_{T\_\bar{x}}\right)}$$

### 2.6. Cell Population-Based Infection and Analysis

Cell population-based experiments were conducted to investigate de novo generation of DI vRNAs. Specifically, we characterized the seed virus (used for infection) and the progeny virions released from the infected cells. For this, confluent MDCK cells in 6-well plates were infected at MOIs of 10 in 250 µL of infection medium. Cells were incubated for 1 h, where the plates were rocked every 20 min to distribute the inoculum evenly. Afterward, the inoculum was removed, cells were washed twice with PBS, and then 2 mL of infection media was added. At 12 hpi, aliquots of supernatants were stored at −80 °C until the purification of the vRNA of the released virus particles. Purification of vRNA of the released progeny virions and of the original seed virus was conducted using the NucleoSpin RNA Virus Kit (Macherey-Nagel, Düren, Germany), according to the manufacturer’s instructions. Afterward, we amplified the complete genome segments using the single-cell RT-PCR method (as described above), but with the following modification: for PCR, we used 5× Phusion HF Buffer (Thermo Scientific), and only 15 PCR cycles were performed. Moreover, besides S1–S3 vRNA, S4–S8 were also amplified (corresponding primers are listed in Table 2). All PCR reaction products of S1–S8 were pooled and then purified using the GeneJET PCR Purification Kit (Thermo Scientific).

A total of 100 ng DNA of amplicon DNA was sheared with the Covaris S2 system (duty cycle 5, intensity 5, 40 s). Sequencing libraries were generated using the KAPA HyperPrep DNA kit (Roche, Rotkreuz, Switzerland): In brief, the fragmented DNA was end-repaired and dA-tailed. Adapters were directly ligated. Adapter-ligated DNA fragments were purified using magnetic beads. Applying three cycles of PCR, the library molecules were amplified using KAPA’s HiFi HotStart ReadyMix. Resulting libraries were again cleaned up and subjected to intense quality controls involving Qubit quantification (Thermo Scientific), Bioanalyzer size assessment (Agilent, Santa Clara, California, U.S.), and qPCR (Roche: KAPA library quantification kit). Sequencing was performed on an Illumina HiSeq2500 system in PE50 mode targeting for two million fragments per library.

### 2.7. Procedure for Single-Cell RNA sequencing (scRNA-Seq)

The single-cell lysates (5 µL) were transferred to a 96-well plate, and subjected to a protocol for Smart-seq2 that allows for the generation of full-length cDNA and sequencing libraries, according to Picelli et al. [48]. Briefly, 0.1 µL of 1:80,000 External RNA Control Consortium (ERCC) RNA spike in controls, 1 µL of dNTPs (10 mM), and 0.1 µL of oligo-dT30VN (5′-AAGCAGTGGTATCAACGCAGAGTACT30VN-3′; 100 µM) were added to the 5 µL of cell lysate (on ice). The mixture was incubated for 3 min at 72 °C followed by 10 min at 10 °C. After hybridization of oligo-dT to the polyA tail, reverse transcription was performed: to each well a master mix containing 0.25 µL RNase-Inhibitor (40 U/µL; final amount/rxn 10 U), 3.4 µL SuperScript First Strand Buffer (5x; final amount/rxn 1x), 0.85 µL DTT (100 mM; final amount/rxn 5 mM), 3.4 µL Betaine (5 M, final amount/rxn 1 M), and 2.04 µL MgCl2 (50 mM; final amount/rxn 6 mM) were added. To start the reverse transcription, at the very last moment, 0.2 µL template switching oligo (TSO: 5′-AAGCAGTGGTATCAACGCAGAGTACATrGrG+G-3′) (100 µM; final amount/rxn 1 µM) and 0.7 µL of SuperScript II reverse transcriptase (200 U/µl; final amount/rxn 140 U) were added and the following incubation program was started in a thermocycler with a heated lid: 90 min of incubation at 42 °C, 10 cycles of 50 °C for 2 min and 42 °C for 2 min, and 15 min of incubation at 70 °C for enzyme inactivation.

Reverse transcription was followed by a preamplification step that was performed on magnetic Agencourt Ampure XP beads (Thermo Scientific): 17 µL of beads was mixed with the RT mix and incubated for 8 min. The plate was put on a magnetic stand for 2 min and the supernatant discarded. To each well, 16 µL of PCR mastermix was added (8 µL of 2x KAPA Hifi Mix, 0.2 µL of ISPCR-primer (5′-AAGCAGTGGTATCAACGCAGAGT-3′, 10 µM) and 8 µL of nuclease free water) and the following PCR program was run: 98 °C for 3 min, 18 cycles of 98 °C for 20 s, 67 °C for 15 s, and 72 °C for 6 min, followed by a final incubation at 72 °C for 5 min.

For clean-up, 16 µL of Ampure XP beads was added and incubated for 8 min. After placement on a magnetic stand, the supernatant was discarded and the beads washed twice with 200 µL of freshly prepared ethanol. Beads were resuspended in 10 µL EB and after further incubation on the magnetic stand, the supernatant containing the DNA was used for library preparation following Illumina’s Nextera XT protocol. 

Therefore, we used 1/5 of the recommended volumes: 2 µL of Tagment DNA (TD) buffer (2x), 1 µL of Amplicon Tagment mix were mixed with 1 µL of the cDNA and incubated for 4 min at 55 °C. Then, 1 µL of NT buffer was added and incubated for 5 min at room temperature. Adapter ligated fragments were barcoded and amplified by adding 3 µL Nextera PCR master mix, and 1 µL of each index 1 and index 2 primers by applying the following cycling protocol: 72 °C for 3 min, 95 °C for 30 s, 15 cycles of 95 °C for 10 s, 55 °C for 30 s, 72 °C for 30 s, and a final incubation of 72 °C for 5 min. Barcoded libraries were pooled and cleaned up using 0.6 volumes of AmpureXP beads. Beads were washed twice with 80% ethanol, eluted in 300 µL EB and a further cleanup was performed by adding an additional 180 µL of beads followed by two washes with 80% ethanol. Beads were resuspended in 100 µL EB. Quality controls were performed involving Qubit quantification (Thermo Fisher), Bioanalyzer size assessment (Agilent), and qPCR (Roche: KAPA library quantification kit). Sequencing was performed on a full lane of the Illumina HiSeq2500 system in PE50 mode.

### 2.8. Data Processing and Quality Control

Gene expression was quantified by Salmon [49], version 0.7.2 including the parameter libType = IU, --posBias and --gcBias. The transcriptome index was built using the Ensembl version 86 Canis familiaris (genome assembly CanFam3.1) cDNA sequences, the genome of IAV of strain PR8, and the sequences of the ERCC RNA spike-ins. For the coverage analysis, STAR (version 2.5.2a) [50] was used in the paired-end and single-end mode, allowing a minimum chimeric segment length of 10 (chimSegmentMin = 10).

Other parameters used for STAR:

-- outFilterMultimapNmax 5
-- outFilterScoreMinOverLread 0.25
-- outFilterMatchNminOverLread 0.25
-- outSJfilterOverhangMin 10 10 10 10
-- outSJfilterCountUniqueMin 1 1 1 1
-- outSJfilterCountTotalMin 1 1 1 1

As a measure of quality control, a sequencing depth of more than 150,000 reads and an ERCC spike-in accuracy of 0.75 was considered. The accuracy was calculated by the Pearson’s correlation coefficient between the known concentration and the measured expression level.

Additionally, samples with at least 10,000 reads mapping to PR8 in the deletion junction analysis were considered.

Salmon quantifies expression level by transcripts per millions (TPM), which includes the ERCC spike-ins. By removing the ERCC spike-ins and scaling the expression values to a million mapped reads, we obtained the expression level from the endogenous transcripts. Genes were filtered out, which were detected (TPM ≥ 1) in less than five samples.

### 2.9. Analysis of Deletion Junctions

Absolute insert sizes of mate pairs mapping to PR8 were extracted from bam files. We calculated the log_2_ ratios between the number of large insert sizes (>1000 bp) and small insert sizes (<=1000 bp) on PR8 S1, S2, and S3 with a pseudocount of 1 × 10^−7^, avoiding the logarithm of zero.

In order to identify the deletion junctions by their exact position, sequence alignment information of split or chimeric reads spanning the junction were used. To obtain the chimeric read information, we first ran STAR using the single-end mode for each read pair separately reducing the alignment artifacts. Next, the two Chimeric.out.junction output files were joined and chimeric reads spanning the junction were counted. Finally, we calculated the deletion junction distance considering the ambiguous split positions by merging chimeric reads with the same distance spanning the same locus with +/− 3 bp difference. Regarding the viral bulk population, junctions were considered that had a distance >1000 bp and covered by >10 reads. For IAV-infected single cell experiments, we included junctions fulfilling the above condition or if junctions were detected in the viral bulk population. Read counts were normalized by counts per millions (CPM).

### 2.10. Data and Software Availability

Data for Figures 2 and 3A,B are available as a supplementary file (Table S1). Collection of next-generation sequencing (NGS) data related to this publication is under BioProject PRJNA590388. The link for the repository that includes the computational analysis is https://github.com/lylamha/influenza_sc. Corresponding files for the analysis (Tables S2–S5) are available as supplementary files.

## 3. Results

### 3.1. Single-Cell Analysis of influenza A virus (IAV)-Infected Cells Demonstrates A Large Cell-to-Cell Heterogeneity in Intracellular Defective Interfering (DI) Viral RNA (vRNA) Content

**Figure 1:**
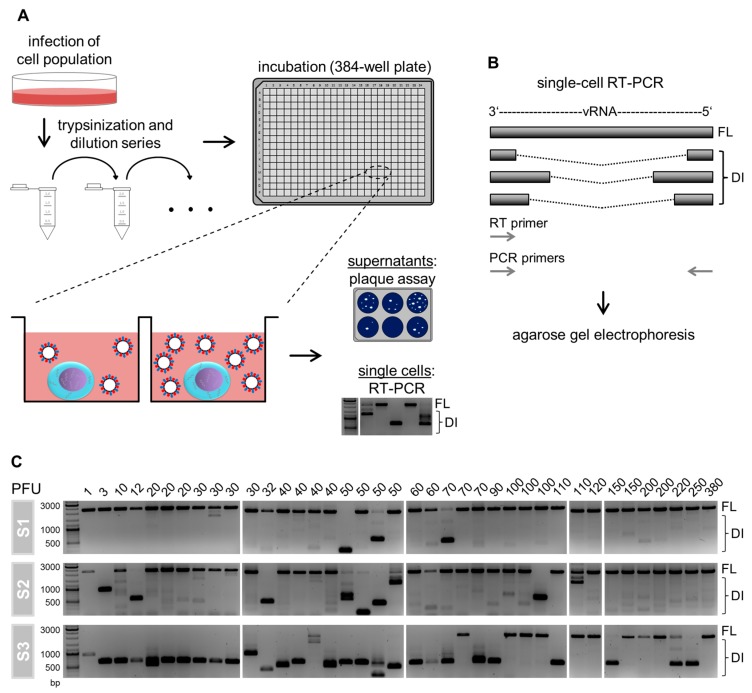
Single-cell analysis approach and cell-to-cell heterogeneity in intracellular defective interfering (DI) viral RNAs (vRNAs). (**A**) Scheme of the experimental procedure. Adherent Madin–Darby canine kidney (MDCK) cells were infected with influenza A virus (IAV) of strain A/PR/8/34 (PR8) at a multiplicity of infection (MOI) of 10. Afterward, the cells were trypsinized, serially diluted, and transferred to a 384-well plate. Single cells in individual wells were identified by phase-contrast microscopy. At 12 hpi, we quantified virus titers in the supernatant of single-cells using the plaque assay. The remaining single cells were lysed and investigated for the presence of intracellular DI vRNAs by reverse transcription polymerase chain reaction (RT-PCR). Illustration adapted from [8,32]. (**B**) Single-cell RT-PCR. DI vRNAs contain large internal deletions of varying lengths and junction points. In RT-PCR, both the RT and PCR primers bind to the terminal 3′ and 5′ ends of vRNA, which results in their complete amplification. In subsequent agarose gel electrophoresis, DI vRNAs occur as fragments of a reduced size in comparison to the FL vRNAs. (**C**) Intracellular FL and DI vRNAs of IAV-infected single cells. The virus yield of single cells is displayed at the top of the gels. The presence of DI vRNAs was investigated on S1, S2, and S3. The DNA ladder is shown at the left. A total of 39 single cells were analyzed of one representative experiment (out of six independent experiments).

To investigate the cell-to-cell heterogeneity of IAV-infected cells with respect to their DI vRNA content, we performed single-cell analysis (Figure 1A). For this, we isolated influenza A/PR/8/34 (PR8)-infected adherent Madin–Darby canine kidney (MDCK) cells (multiplicity of infection (MOI) of 10) in 384 well plates utilizing a limiting dilution approach, as described previously [8,32]. At 12 h post infection (hpi), the virus yield in the supernatant of single cells was quantified using a plaque assay. Moreover, the single cells were investigated for the presence of intracellular DI vRNAs via RT-PCR. Here, the RT and PCR primers hybridize to the terminal end of vRNAs, which facilitates their complete amplification in RT-PCR (Figure 1B). Thus, the resulting PCR products of the FL and DI vRNA can be discriminated in agarose gel electrophoresis based on their sizes.

Figure 1C shows the content of DI vRNAs of S1, S2, and S3 of IAV-infected single cells. Surprisingly, we observed a large cell-to-cell diversity in intracellular DI vRNAs. Specifically, the between-cell heterogeneity comprised differences regarding (i) the presence or absence, (ii) the signal intensity, (iii) the length (in bp) of the DI vRNAs, and (iv) the genome segment on which DI vRNAs occur. Please note that only S1–S3 were investigated as these segments typically bear deletions [11,12,13]. Furthermore, it appeared that single cells showing a virus yield of ≥60 plaque-forming units (PFU) contained less frequently DI vRNAs overall, which was most apparently observed for S3. Likewise, we observed DI vRNAs on S2 more frequently in cells that displayed a virus titer <60 PFU. Concurrently, FL vRNAs were more frequently observed (for S3) for single cells that released ≥60 PFU. The absence of a signal corresponding to the FL vRNA in some single cells most likely indicates a concentration that was below the limit of detection of the RT-PCR assay established. Please also note the presence of faint signals of DI vRNAs, which either suggest low levels, or de novo generated DI vRNAs at the single-cell level. Occasionally, we also observed high-productive IAV-infected single cells that nevertheless contained DI vRNAs. These cells may indicate rarely occurring defective, non-interfering vRNAs [11,25], or additional unknown factors that can affect the single-cell virus yield beyond DI vRNAs. Taken together, we show that IAV-infected single cells display a vast diversity in intracellular DI vRNAs.

### 3.2. Infected Single Cells with a Low Progeny Virus Titer Show a High Load of Intracellular DI vRNAs

**Figure 2:**
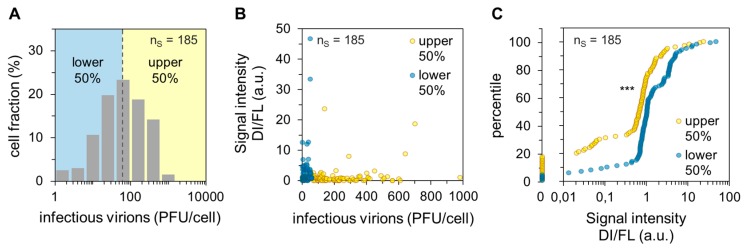
Dependency of the single-cell virus yield on intracellular DI vRNAs. Single PR8-infected MDCK cells (MOI = 10) were investigated for the presence of DI vRNAs on S1–S3 by single-cell RT-PCR and released virus yields by plaque assay at 12 hpi (as shown Figure 1). The pooled data of six independent experiments are shown. n_S_ indicates the number of single-cell measurements. (**A**) Histogram of the single-cell virus yield. Dashed line indicates the median of virus titer (60 plaque-forming units (PFU)). (**B**) Dependency of the virus yield on the quantity ratio of DI to FL vRNAs. The signal intensities of the DI and FL vRNAs from agarose gels were quantified using ImageJ. Afterward, the quantity ratios were calculated by dividing the sum of signal intensities of the DI vRNAs on S1–S3 by the sum of the signal intensities of the FL vRNAs on S1–S3. (**C**) Percentile graph of the data shown in (B). The data were divided into 100 quantiles. ***, *p* < 0.0005 by the Wilcoxon rank sum test.

Next, we quantified the results from single-cell RT-PCR for statistical analysis. For this, we performed six independent experiments yielding 185 single-cell measurements. The corresponding histogram of virus yield is displayed in Figure 2A. In addition, we used the image processing program ImageJ (National Institutes of Health, Bethesda, Maryland, USA) to quantify the signal intensities of DI and FL vRNAs of the images obtained from agarose gel electrophoresis.

Subsequently, we calculated the quantity ratio of DI to FL vRNAs for each cell by dividing the sum of signal intensities of DI vRNAs on S1–S3 by the sum of signal intensities of the FL vRNAs on S1–S3. Note that a low level of FL vRNAs typically coincided with a high load of DI vRNAs (see also Figure 1C), most likely since the DI vRNAs inhibit the synthesis of the cognate FL vRNAs [11]. Figure 2B shows the dependency of the quantity ratio of DI to FL vRNAs on the single-cell virus titer. It appeared that the lower 50% of cells (regarding the cell-specific virus yield, “lower”) showed an overall higher quantity ratio. In agreement with our previous observations (Figure 1C and Figure 2B), statistical analysis of the dataset revealed a significant difference between the “upper” and “lower” cells regarding the quantity ratio of DI to FL vRNAs (shown by the Wilcoxon rank sum test, *p* < 0.001). This difference is visualized in the corresponding percentile graph (Figure 2C). Here, for instance, 80% of the “upper” cells showed a quantity ratio of DI to FL vRNA of ~1.3 or less, while 80% of the “lower” cells showed a ratio of ~3.4 or less. In summary, we show that a low single-cell virus yield is connected with a high DI vRNA content, and vice versa. Therefore, DI vRNAs appear to be a factor that can affect the cell-to-cell heterogeneity of IAV replication.

### 3.3. scRNA-Seq Reveals a Decrease of Host Cell mRNA Fraction and an Increase of Viral mRNA Fraction in High-Yield Cells

**Figure 3:**
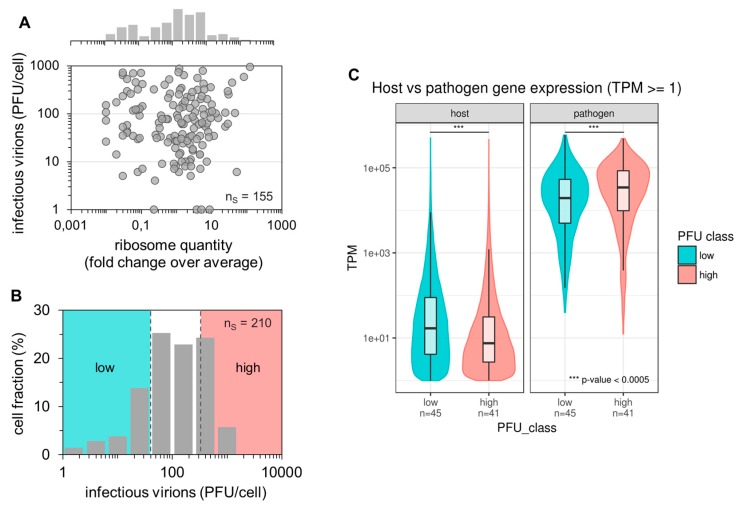
Impact of ribosome quantity, fraction of viral and host cell mRNAs on single-cell virus yield. Single PR8-infected MDCK cells (MOI = 10) were incubated until 12 hpi (as shown in Figure 1). Afterward, virus yields were investigated using the plaque assay, intracellular 18S rRNA via real-time RT-qPCR, or intracellular host cell and viral mRNA via scRNA-seq. n_S_ indicates the number of single-cell measurements. (**A**) Dependency of single-cell virus yield on the ribosome content. The ribosome quantity was estimated based on the assumption that exactly one 18S rRNA molecule is associated with each ribosome. Next, the fold change over the average single-cell 18S rRNA expression was calculated. The pooled data of multiple independent experiments are shown (*n* = 4). (**B**) Histogram of the single-cell virus yield of cells investigated by scRNA-seq (panel (C)). Colors indicate the lower and upper 22.86% of cells with respect to the virus yield (“low” and “high”, respectively). The pooled data of multiple independent experiments are depicted (*n* = 3). (**C**) Host cell and viral mRNA content. Expression values (TPM ≥ 1) for host and viral genes are plotted on a log-scale for cells classified into low and high virus yields, as shown in Figure 3B. ***, *p* < 0.0005 by the Wilcoxon rank sum test.

Sequencing library preparation protocols for scRNA-seq typically involve a step that removes ribosomal RNAs (rRNAs) [48]. As we hypothesized an impact of the ribosome content on single-cell virus replication, we performed real-time RT-qPCR of single-cell lysates to evaluate the rRNA levels. Specifically, we measured the 18S rRNA, which is bound to the small ribosomal subunit at exactly one molecule per ribosome [47]. Afterward, we calculated (for each single cell) the fold change over the average single-cell expression of 18S rRNA. Just like non-infected cells (Figure S5A), we observed a huge cell-to-cell variability in the 18S rRNA content in infected cells, with differences in rRNA quantities that spanned more than four orders of magnitude (Figure 3A). However, we did not find a significant correlation between the ribosome content and the single-cell virus yield.

In order to identify additional factors that can contribute to the large cell-to-cell heterogeneity in IAV replication, we next performed whole transcriptome analysis by scRNA-seq using Illumina-based next-generation sequencing (NGS) technologies. Although similar studies have been conducted previously [37,43,44] including IAV-infected cells [39,40], only the viral mRNA level was used as a read-out to evaluate virus replication. In contrast, we also quantified the extracellular progeny virus yields, and thus, anticipated additional insights. To generate samples for scRNA-seq, we conducted three independent single-cell infection experiments yielding 210 single-cell samples. The corresponding distribution of the virus titer is shown in Figure 3B. Here, we defined the “high” and “low” productive single cells based on the upper and lower 22.86% of cells (yielding each 48 samples) regarding their virus yield, respectively. Virus titers ranged from 1–40 and 330–1100 PFU for “low” and “high” productive cells, respectively. The single-cell lysates were then subjected to scRNA-seq.

Quality filtering based on library size and External RNA Control Consortium (ERCC) spike-in accuracy yielded 86 single cells (45 low and 41 high productive cells) suitable for further analysis (Figure S1). Furthermore, we filtered out genes that were detected (transcripts per million (TPM) ≥ 1) in less than five samples, leading to a set of 2755 host cell genes. The remaining cells had an average host gene detection rate of 1381 genes (median 845 genes) per cell, which is an overall low detection rate in scRNA-seq [51,52,53]. This may be explained by the high viral transcriptional activity, likely suppressing host cell gene expression and dominating the cellular transcription by more than three orders of magnitude (Figure 3C and Figure S5B). Interestingly, high-productive cells showed a significantly lower fraction of host cell mRNAs and significantly higher fraction of viral mRNAs compared to low-productive cells (Wilcoxon rank sum test *p* < 2.2 × 10^−16^ for the host and *p* = 8.23 × 10^−5^ for viral mRNA). In agreement with this, we observed a negative correlation between the host cell mRNA level and the virus titer, and a positive correlation between the viral mRNA level and the virus yield (Figure S5C,D). Unfortunately, the signal for host cell expression was too low and noisy to perform reliable differential expression analysis for the identification of potential marker genes affecting IAV replication (Figure S2). In contrast, the signal for virus-derived mRNAs was strong enough to allow for further analysis (Figure 3C and Figure S2). Taken together, we observed no correlation of the ribosome content of single cells with the virus titer. Moreover, scRNA-seq analysis showed that fractions of viral mRNAs were increased, and those of host cell mRNAs decreased in high-yield cells compared to low-yield cells. 

### 3.4. scRNA-Seq Analysis Reveals an Association of the DI mRNA Content and the Single-Cell Virus Yield

**Figure 4:**
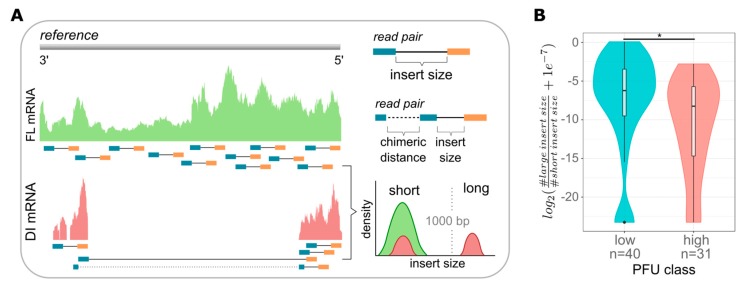
Computational workflow for the analysis of DI mRNAs and dependency of DI mRNAs on single-cell virus yield. At 12 hpi, single PR8-infected MDCK cells (MOI = 10) were investigated for released virus yields by the plaque assay (as shown in Figure 1) and the presence of DI mRNAs on S1–S3 by scRNA-seq. (**A**) Next-generation sequencing (NGS)-based computational workflow to detect DI mRNAs. Read pairs were mapped to the influenza reference genome. The estimated insert sizes were gathered from the alignment files. Typically, in the absence of structural variation, read pairs have an expected average insert size of about 250 nucleotides, which refers to FL mRNA. However, in the presence of large internal deletions, the insert size is larger than 1000 nucleotides, which refers to DI mRNAs. Chimeric reads spanning the deleted region can be used to locate the position of the deletion with its actual length. (**B**) Ratio of DI to FL mRNAs in cells classified as “low”- and “high”-yield single cells (as shown in Figure 3B). The log_2_-ratio between the number of large insert sizes and short insert sizes was used and a pseudo-count of 1e-7 was added to avoid a log of zero. Colors indicate the lower and upper 22.86% of cells with respect to the virus yield (“low” and “high”, respectively). *, *p* < 0.01 by the Wilcoxon rank sum test. The pooled data of multiple independent experiments are depicted (*n* = 3).

DI vRNAs typically retain the terminal 3′ and 5′ ends harboring the promoter regions, which are necessary for viral transcription [11,12]. Therefore, the majority, if not all, DI vRNAs are able to transcribe and generate DI mRNAs [54,55,56]. Thus, we continued analyzing DI mRNAs by scRNA-seq. Indeed, using sequence analysis, we observed a high heterogeneity in the coverage profiles of viral mRNAs, in particular for S3, which may indicate the presence of DI mRNAs (Figure S3). For a more detailed analysis, we developed a computational workflow (similarly seen elsewhere [39,57]) as illustrated in Figure 4A, aiming to pinpoint individual DI mRNAs. Briefly, paired-end reads were mapped against the influenza genome providing an estimate of the distance (insert size) of the mate pairs. We used an insert size cut-off of 1000 bp to distinguish between short and long insert sizes representing FL transcripts and deletions, respectively. Furthermore, chimeric reads allowed the breaking point of the deletions to be located and were used as an additional but not necessary condition to indicate the presence of DI mRNAs. In agreement with previous reports [11,12,13], we have only scarcely detected putative DI mRNAs on S4–S8, and thus, focused on the analysis of S1–S3.

Using this workflow, we quantified the expression of DI and FL mRNAs and determined their quantity ratio (Figure 4B). In line with our previous results (Figure 2), we observed that low-yield cells showed a significantly higher ratio of DI to FL mRNAs compared to high-yield cells (Figure 4B and Figure S4). Interestingly, we also found low-yield cells with no detectable DI mRNAs (as indicated by the outliers (five cells in total) in the left violin plot at the bottom, Figure 4B). On the other hand, some high-productive cells nevertheless contained DI vRNAs (Figure 1C, lower right corner). Such rare cells likely indicate additional factors that can affect the single-cell virus yield, besides DI mRNAs or DI vRNAs. In summary, scRNA-seq analysis confirmed that the load of intracellular DI mRNAs, derived from DI vRNAs, most likely affects the virus release of IAV-infected single cells.

### 3.5. De Novo Generation of DI vRNAs Observed at the Single-Cell Level

**Figure 5:**
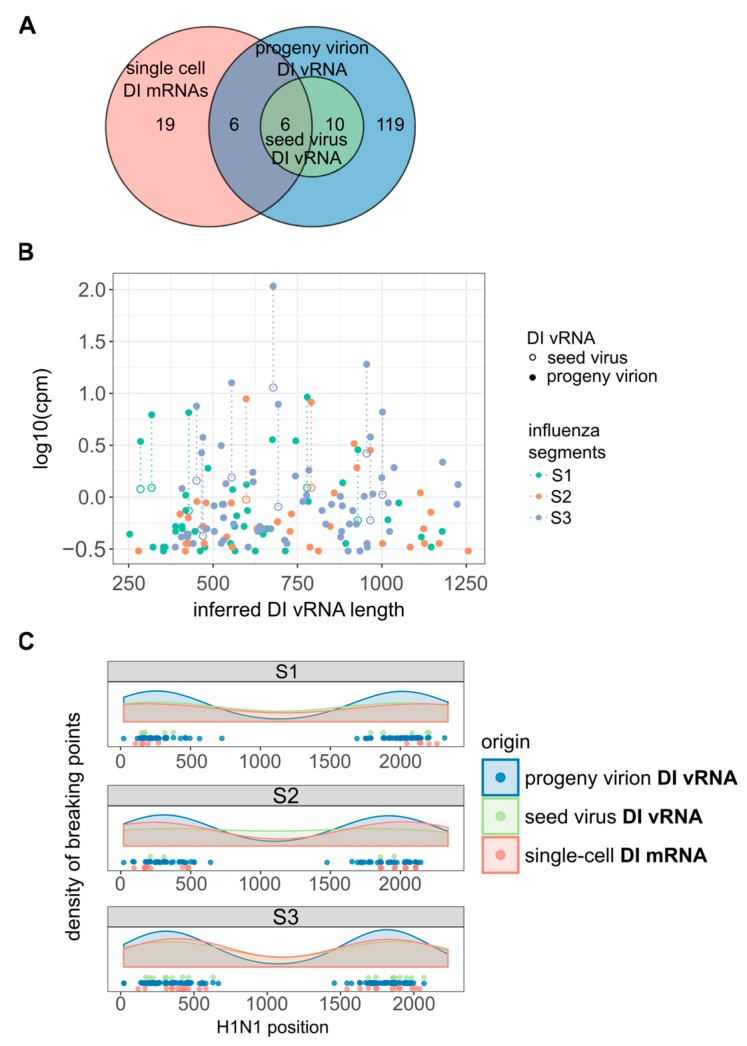
De novo generation of DI vRNAs. De novo generation was studied at the population and single-cell level. MDCK cells were infected with PR8 virus at an MOI of 10, and incubated until 12 hpi for cell population-based studies, or cells were trypsinized to facilitate single cell isolation (as shown in Figure 1) and incubation until 12 hpi. Seed virus vRNAs and vRNAs of progeny virions (produced in cell population-based infections) were analyzed by bulk RNA-seq. Single-cell intracellular viral mRNAs were investigated via scRNA-seq. Chimeric reads spanning the deleted region were used to identify the distinct DI vRNAs and DI mRNAs and to locate the deletion junction region (as shown in Figure 4A). The pooled data of multiple independent experiments are depicted (*n* = 2 for population- and *n* = 3 for single cell-based experiments). (**A**) Venn diagram showing the number of distinct DI vRNAs in the seed virus and progeny virions, and of DI mRNAs in single cells. (**B**) Diversity in DI vRNA sizes in the seed virus and progeny virions and their expression level. The expression level of DI vRNAs was approximated by the normalized number of chimeric reads spanning the deletion junction. (**C**) Distribution of deletion junctions of DI vRNAs and DI mRNAs. Start and end position of the deletion junction are marked on the corresponding segment.

**Figure 6:**
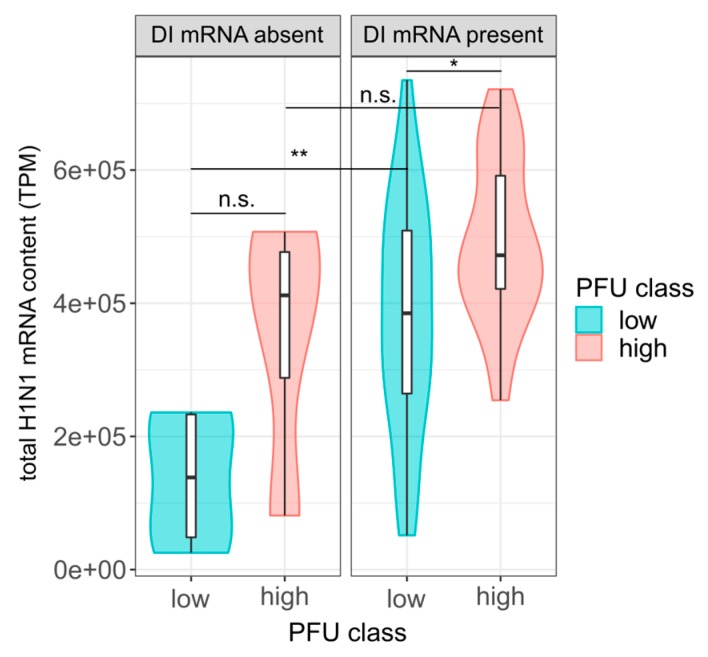
Influence of total viral mRNAs and DI mRNAs on the single-cell virus yield. At 12 hpi, single PR8-infected MDCK cells (MOI = 10) were investigated for virus yield by plaque assay (as shown in Figure 1), the total viral mRNA content and the presence of intracellular DI mRNAs on S1–S3 by scRNA-seq. Single cells were subdivided into DI mRNA positive and negative cells, according to the presence and absence of large insert sizes (>1000 bp), as shown in Figure 4A. Total viral mRNA content in transcripts per million (TPM) was calculated by removing read pairs with a large insert size. Colors indicate the lower and upper 22.86% of cells with respect to the virus yield (“low” and “high”, respectively), as shown in Figure 3B. Sample sizes from left to right (*n* = 5, 4, 35, 27). *, *p* < 0.05; **, *p* < 0.005; n.s., *p* > 0.05, not significant (by the Wilcoxon rank sum test). The pooled data of multiple independent experiments are depicted (*n* = 3).

Next, we studied the de novo generation of DI vRNAs in single cells as well as in cell population-based samples. Therefore, we first compared the seed virus used for infection with the progeny virions produced in an infection using bulk RNA-seq. In addition, we investigated DI mRNAs, derived from de novo generated DI vRNAs, in single-cell infections using scRNA-seq. Note that all bulk- and single-cell infection conditions were identical in this study (Figure 1, Figure 2, Figure 3, Figure 4, Figure 5 and Figure 6).

Using the chimeric reads as a marker to identify different DI vRNAs, we observed 16 distinct DI vRNAs in the seed virus (Figure 5A). Strikingly, the number of different DI vRNAs increased to 141 in the progeny virions, implicating 125 de novo generated DI vRNAs. Figure 5B shows the inferred lengths of DI vRNAs with respect to the expression level of DI vRNAs. We approximated the expression level of DI vRNAs by the normalized number of chimeric reads spanning the deletion junction. However, the number represents a lower bound for the expression level as read pairs mapping beyond the deletion junction cannot be technically distinguished between FL and DI vRNAs. We observed that DI vRNAs pre-existing in the seed virus became more abundant in the progeny virions, suggesting that they were amplified within a single cycle of high MOI infection, as to be expected for DIP propagation [11]. Accordingly, the apparently de novo generated DI vRNAs showed lower expression levels in the progeny virions. Moreover, all DI vRNAs contained breaking points situated ~250–400 bp apart from the terminal ends of S1–S3 (Figure 5C), which is in accordance with previous observations [57,58,59].

Interestingly, utilizing scRNA-seq analysis, we observed deletion junctions of DI mRNAs in infected single cells at similar regions (Figure 5C), which seems to confirm their identity as DI mRNAs arising from DI vRNAs. Moreover, we observed six deletion junctions in the single-cell DI mRNAs that were identical to those identified in the DI vRNAs of the seed virus (Figure 5A), indicating the amplification of these pre-existing DI vRNAs (and thus, DI mRNAs) in single cells. Furthermore, we also detected 25 deletion junctions not observed in the seed virus, implicating the de novo generation of DI vRNAs that can be observed at the single cell level. In agreement with this, we also observed such apparent de novo generated DI vRNAs in single cells by RT-PCR analysis in Figure 1C, as indicated by the presence of faint signals corresponding to DI vRNAs. However, also note the presence of six single-cell DI mRNA deletion junctions that were identical to those observed for apparently de novo generated DI vRNAs present in the progeny virions of the bulk infection (Figure 5A). Such an observation most likely relates to low levels of pre-existing DI vRNAs in the seed virus that were not detectable. Nevertheless, our results strongly indicate the generation of highly diverse de novo DI vRNAs at the single-cell level, which unveils an additional facet of the large cell-to-cell heterogeneity in IAV replication.

### 3.6. Viral mRNA Level and DI mRNA Content Are Both Connected with the Single-Cell Virus Yield, Independently from One Another

As described above, a high load of viral mRNAs (Figure 3C) and a low content of DI vRNAs and DI mRNAs (Figure 2 and Figure 4) was observed in high-yield single cells. We next investigated whether the two factors can both, independently from one another, affect single-cell virus titers. For this, we subdivided cells that contained or did not contain DI mRNAs based on the presence and absence of large insert sizes (indicating DI mRNAs). Subsequently, we investigated whether in each group, a higher level of viral mRNA content could be observed for high-yield cells compared to low-yield cells. For this, we subtracted all read pairs with a large insert size (indicating DI mRNA) from the total viral mRNA content.

Indeed, we observed higher levels of viral mRNA in high-yield cells in each group of cells that were either negative or positive in DI mRNAs (Figure 6). However, this difference was only significant in the group of cells that were positive in DI mRNAs, and not in cells negative in DI mRNAs. This might be explained by the low sample size in the latter group, which may have not provided enough statistical power. Nevertheless, it appeared that regardless of whether DI mRNAs were present or not, the total level of viral mRNA was correlated with the single-cell virus yield.

Conversely, in the group of low-yield cells, we observed a subset of cells that nevertheless contained relatively high levels of total viral mRNAs. Intriguingly, these cells tested positive regarding the presence of DI mRNAs. These results suggest that, despite higher levels of viral mRNAs, the presence of DI mRNAs (indicative for DI vRNAs) likely resulted in interference with virus replication, and thus, a decrease in virus yield. Although not significant, a similar trend was observed in the group of high-yield cells where the cells containing a higher level of mRNAs, in turn, also tested positive for DI mRNAs. We concluded that the DI mRNA content and the level of viral mRNAs are both, independently from one another, connected with single-cell virus production.

## 4. Discussion

The origin of the large cell-to-cell heterogeneity in virus infections is still not fully understood [29,30,31,32,34,35,36,37,38,39,40,43,44]. We conducted single-cell analysis of IAV replication and reveal an association of DIP co-infections and single-cell progeny virus yields. Thus, differences in the content of DI vRNAs and DI mRNAs (observed between individual cells) may well contribute to the large cell-to-cell heterogeneity in virus release. However, we also found a few exceptions from this observation, which may be explained by scarcely occurring DI vRNAs, which cannot interfere with standard virus (STV) replication for unknown reasons [11,25], or more likely, the presence of additional factors that can influence single-cell virus yield.

Furthermore, we observed a multifaceted single-cell variability in intracellular DI vRNAs and DI mRNAs that involved (i) the presence or absence, (ii) the load and (iii) the length, and (iv) the genomic segments on which the DI vRNAs and DI mRNAs occurred. Such a large heterogeneity may well reflect the virus-to-virus diversity in the DI vRNA content of individual DIPs in the infecting virus population. Alternatively, some DI vRNAs might have originated de novo during the infection. In particular, our results of single-cell RT-PCR and subsequent agarose gel electrophoresis (Figure 1C) and of scRNA-seq (Figure 5) indicate de novo generation of DI vRNAs, observed at the single-cell level. This hints at an additional facet of the large cell-to-cell heterogeneity in IAV replication. 

Previously, single-cell virus replication under the influence of DIP co-infections has been studied in vesicular stomatitis virus (VSV)-infected cells [60,61] and IAV-infected cells [39], however, the latter study focused on the effect of DIP co-infections on the host cell response. In particular, an association between defective viral genomes and the stimulation of the innate immune response was observed [39]. However, a direct correlation of the load of intracellular DI RNAs with single-cell progeny virus release was not investigated. The same is true for previous studies involving single-cell VSV infections [60,61]. Here, the authors instead co-infected VSV-infected cells with different multiplicities of DIP (MODIP). In line with our results, the authors observed a reduction and delay in VSV replication once co-infected with increasing MODIP [60,61].

Upon infection, IAVs hijack the cells’ biosynthetic machinery including ribosomes. More specifically, ribosomes are recruited for the translation of viral proteins that are necessary for virus propagation. Previously, a correlation between cellular resources required for protein synthesis and virus release was suggested for the T7 phage infection [62]. Interestingly, we observed a vast cell-to-cell diversity in the ribosome content of IAV-infected MDCK cells with quantitative differences that spanned roughly four orders of magnitude. Surprisingly, however, a correlation between the single-cell virus release and the ribosome content was not observed. This indicates that ribosomes are sufficiently present in each individual cell, despite the large quantitative between-cell variability, and that they do not pose a bottleneck in IAV production.

Furthermore, we showed that high-yield cells harbor an elevated fraction of viral mRNAs as compared to low-yield cells. This observation is in agreement with a previous study, where we observed elevated levels of vRNAs in IAV-infected single cells showing high virus titers [32]. In this context, it may be conclusive that a higher number of vRNAs leads to an elevated transcription of viral mRNAs, which, in turn, provide more templates for an enhanced translation of viral proteins that are required for virus particle production. Conversely, we found that the fraction of host cell mRNAs was decreased in high-yield cells. This was similarly observed for herpes virus-infected single cells [63]. Here, a higher cellular gene expression coincided with lower viral gene expression. It was speculated that specific host cell determinants are responsible for a supposed suppression of virus replication. Such determinants may be the innate immune response [36,37,38,39], and other antiviral host cell factors that were previously identified by scRNA-seq [37,39,40,43,44].

However, the concentration of host cell mRNAs of the single-cell samples was very low. Thus, the measurements were affected by a high degree of technical noise, preventing the accurate identification of host cell transcripts that are specific for high- or low-yield cells. Note that in other studies, host cell mRNAs of virus-infected single cells could be reliably identified [37,39,40,43,44]. We suppose that the following technical and experimental differences of our study are responsible for the noisy data. First, a relatively high volume (5 µL) of single-cell lysates was investigated, leading to low analyte concentrations and thus, decreased sensitivities when compared to the volumes (in the pico- and nanoliter range) that can be achieved with the microfluidics-based technologies [64] used in the above-mentioned studies. Second, we used an open single-cell cultivation system in 384-well plates, which may be susceptible to contamination with ubiquitously present ribonucleases [65]. Third, highly permissive MDCK cells were used, which allow for strong virus replication and high virus titers [66], but presumably, low cellular gene expression levels. Finally, experiments were performed at high MOI, leading to a strong virus replication and likely, an enhanced suppression of host cell gene expression.

Nevertheless, the expression of viral mRNAs was high enough for the accurate analysis of DI mRNAs by scRNA-seq. Note that the results of bulk RNA-seq indicated similar junction regions of the DI vRNAs in comparison to the single-cell DI mRNAs (~250–400 bp apart from the terminal ends, in agreement with previous observations [57,58,59]), which confirms their identity as transcriptional descendants of the DI vRNAs. Such DI mRNAs, which can result in the translation of small polypeptides, were described previously for IAV-infected cells [54,55,56]. It was long speculated that some of these polypeptides may have a function, like contributing to the defectiveness or interference of a DIP [55,56]. Moreover, it was shown that such small polypeptides can act immune–stimulatory and thus, can contribute to the antiviral activity of DIPs [54]. Finally, our results suggest that the level of viral mRNAs and the load of DI mRNAs are both, independently from one another, connected with single-cell virus yield.

Taken together, our results underline that elucidation of the origins of the high cell-to-cell heterogeneity in virus replication is a complex multi-parametric problem, where many different factors each exert an individual effect. In particular, we showed that DI vRNAs and DI mRNAs (interfering with virus replication), total level of viral mRNAs (facilitating the production of viral proteins), the fraction of host cell mRNAs (of which some factors may inhibit virus replication), but not the ribosome content, may have an impact on yields of a single IAV-infected cell. Nevertheless, it seems that various additional determinants remain to be elucidated. Overall, single-cell virology qualifies as a valuable tool to broaden our understanding of the vast cell-to-cell variability in virus replication.

## Figures and Tables

**Table 1:** Primers for single-cell reverse transcription polymerase chain reaction (RT-PCR).

Reaction	Target	Primer Name	Sequence (5′ -> 3′)
RT	All segments	Uni12	AGCAAAAGCAGG
PCR	Segment 1	S1 Uni for	AGCGAAAGCAGGTCAATTAT
		S1 Uni rev	AGTAGAAACAAGGTCGTTTTTAAAC
	Segment 2	S2 Uni for	AGCGAAAGCAGGCAAACC
		S2 Uni rev	AGTAGGAACAAGGCATTTTTTCATG
	Segment 3	S3 Uni for	AGCGAAAGAAGGTACTGATCC
		S3 Uni rev	AGTAGAAACAAGGTACTTTTTTGGAC

**Table 2:** Primers for RT-PCR (S4–S8).

Reaction	Target	Primer Name	Sequence (5′ -> 3′)
PCR	Segment 4	S4 Uni for	AGCAAAAGCAGGGGAA
		S4 Uni rev	AGTAGAAACAAGGGTGTTTT
	Segment 5	S5 Uni for	AGCAAAAGCAGGGTAGATAATC
		S5 Uni rev	AGTAGAAACAAGGGTATTTTTC
	Segment 6	S6 Uni for	AGCGAAAGCAGGGGTTTAAAATG
		S6 Uni rev	AGTAGAAACAAGGAGTTTTTTGAAC
	Segment 7	S7 Uni for	AGCGAAAGCAGGTAGATATTG
		S7 Uni rev	AGTAGAAACAAGGTAGTTTTTTAC
	Segment 8	S8 Uni for	AGAAAAAGCAGGGTGACAAA
		S8 Uni rev	AGTAGAAACAAGGGTGTTTT

## Supplementary Materials

The following are available online at https://www.mdpi.com/1999-4915/12/1/71/s1, Figure S1: Quality control; Figure S2: Technical variability; Figure S3: Coverage plot of PB2, PB1 and PA of example cells; Figure S4: Amount of DI RNAs affecting the virus titer; Figure S5: Controls for and statistical analyses on the impact of ribosome quantity, fraction of viral and host cell mRNAs on single-cell virus yield (related to Figure 3); Table S1: Data of Figure 2 and Figure 3A,B; Table S2: Single-cell gene expression after quality control (QC); Table S3: Sample annotation after QC; Table S4: Small and large insert size counts; Table S5: Identified and annotated chimeric read junctions.
