# Supplementary material for: Single-Cell Analysis Uncovers a Vast Diversity in Intracellular Viral Defective Interfering RNA Content Affecting the Large Cell-to-Cell Heterogeneity in Influenza A Virus Replication
Source: Viruses. 2020 Jan 7;12(1):71. doi: 10.3390/v12010071 (PMC7019491; doi:10.3390/v12010071)
Supplement: Supplementary file 1 [file viruses-12-00071-s001.zip › Figure S1-S5.pdf]

Supplementary Figures

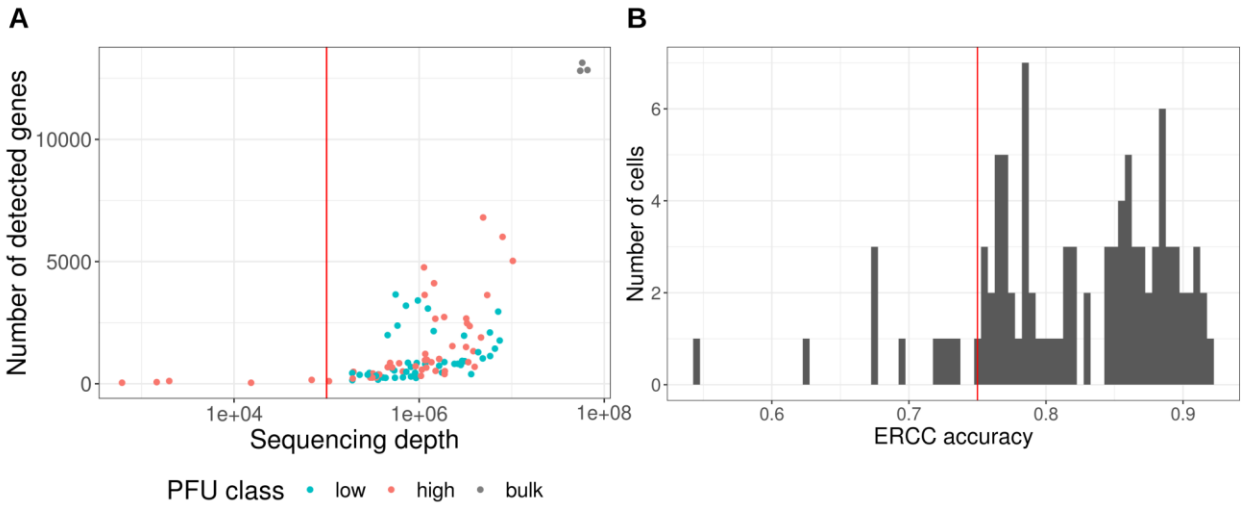

Figure S1. Quality control. (**A**) Gene detection rate (TPM >=1) for 95 cells and reference bulk RNA-seq experiment dependent on sequencing depth. Cells with a sequencing depth of less than 150,000 reads were removed from further analysis. (**B**) ERCC accuracy in single cells. ERCC accuracy were obtained by Pearson correlation coefficient between measured TPM level and known concentration level within a cell. Cells with a lower ERCC accuracy of 0.75 were removed.

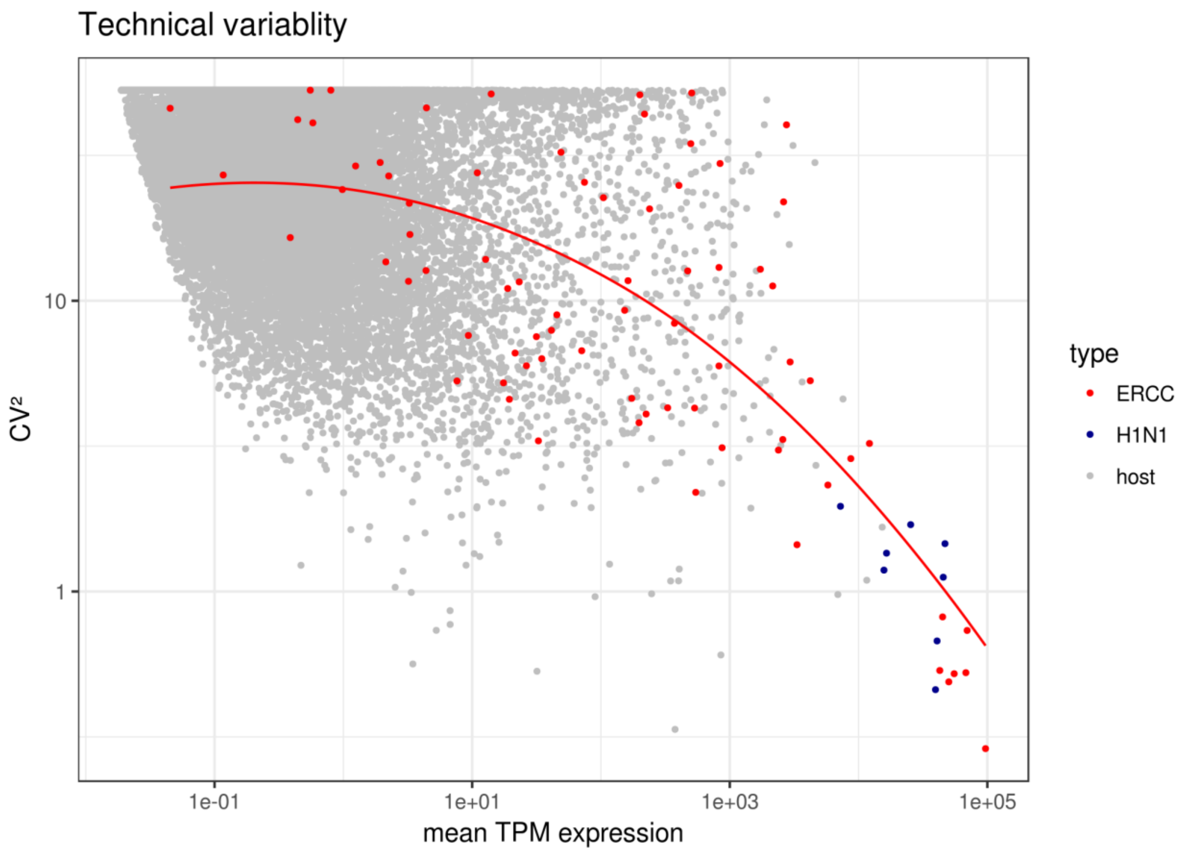

Figure S2. Technical variability. Mean TPM expression and squared coefficient of variation (CV^2) were calculated from 86 samples (after QC: > 150,000 reads and ERCC accuracy > 0.75) and ~20K genes (detected in any cell). Endogenous host cell genes are grey, ERCC spike-ins red and IAV genes are blue-coloured. Red line indicates the fitted line of measured ERCC spike-in expression representing the technical variability across the experiment. Plot shows high noise level (variability) in lowly expressed transcripts. No host cell transcripts go beyond technical variability.

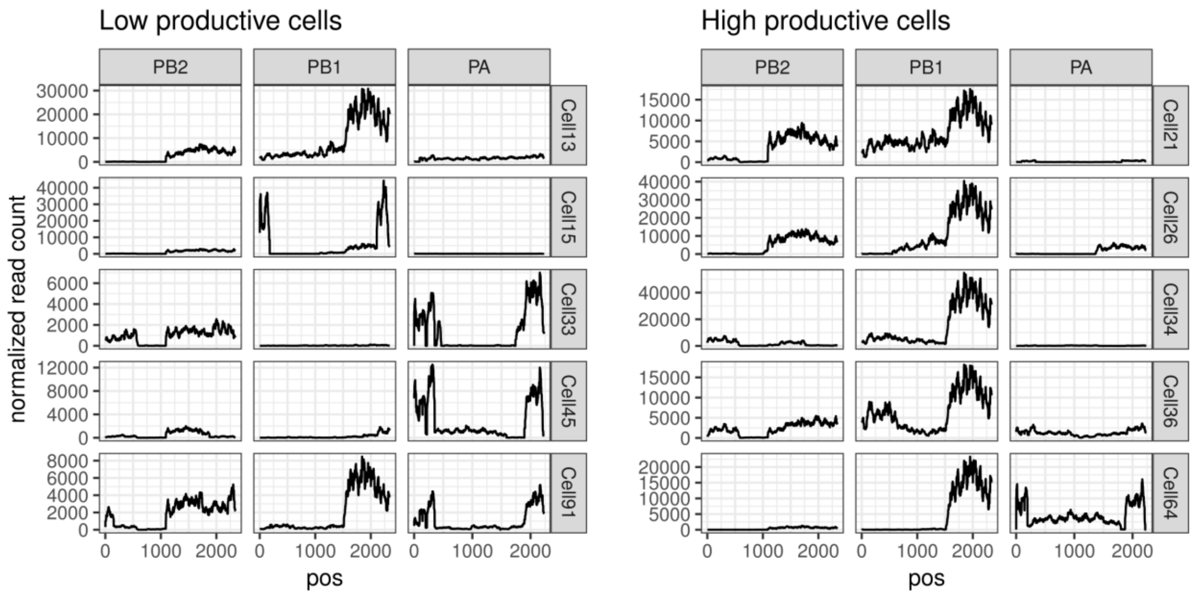

Figure S3. Coverage plot of PB2, PB1 and PA of example cells. Cells show heterogeneity in coverage tracks in IAV genes PB2, PB1 and PA between low- and high-yield cells. High transcription of flanking regions on the segment indicates large internal deletion present in PB1 and PA. Read counts were normalized by million mapped reads.

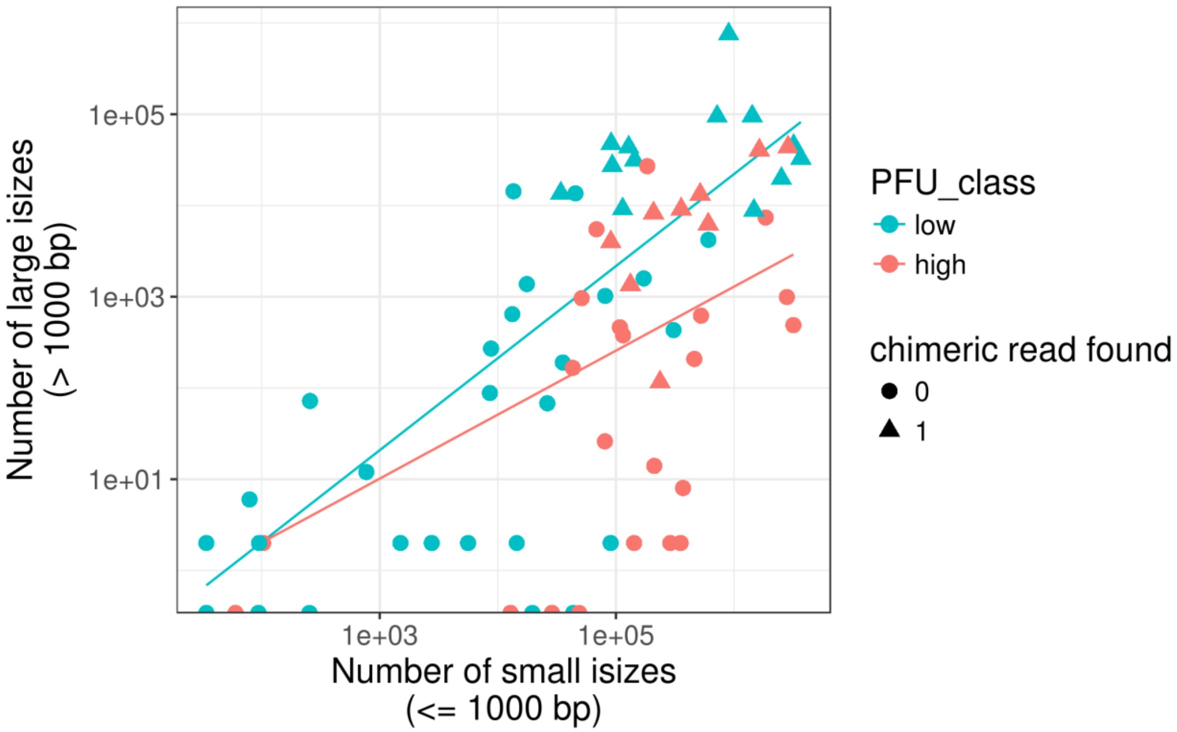

Figure S4. Amount of DI RNAs affecting the virus titer. Low-yield cells show a higher amount of internal deletions than full length transcripts as an estimate of the insert size. Insert sizes with a distance of > 1000 bp are considered large, and <= 1000 bp are considered small. Chimeric reads spanning the junction can be found in cells with a high coverage.

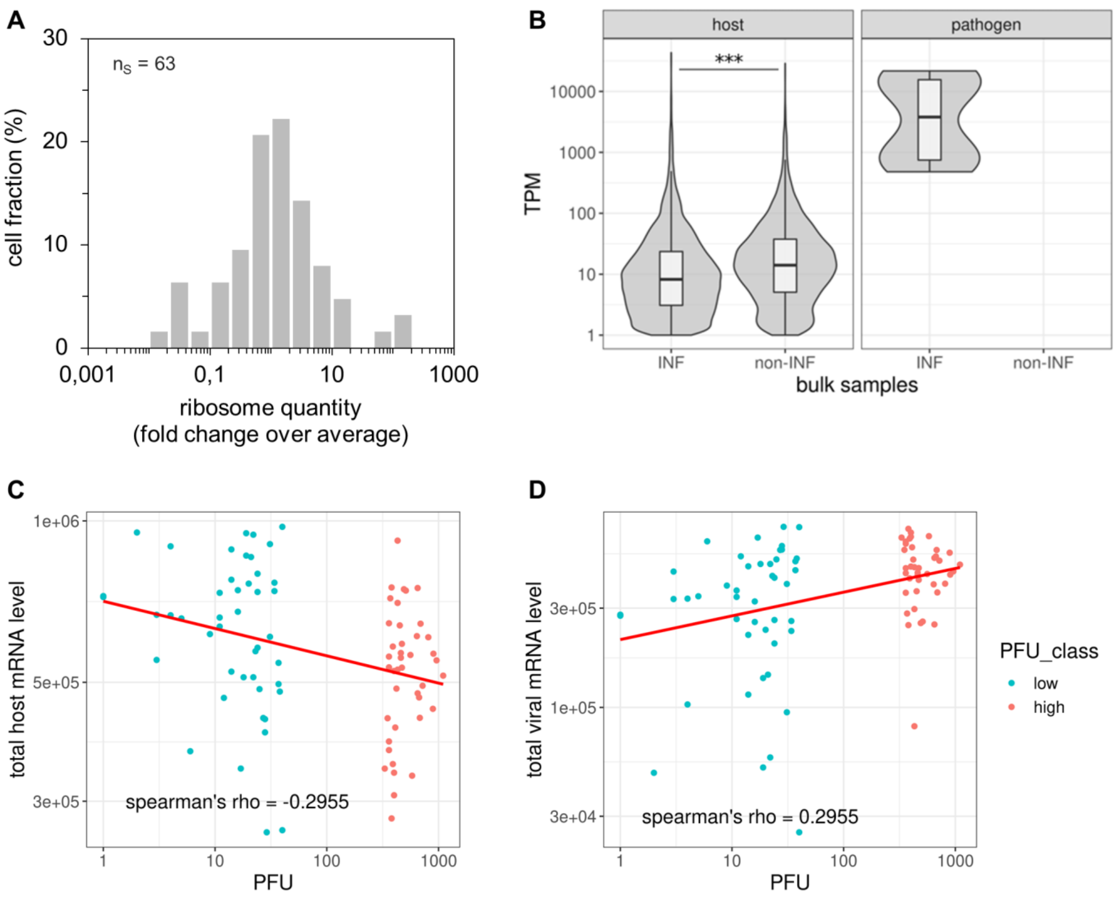

Figure S5. Controls for and statistical analyses on the impact of ribosome quantity, fraction of viral and host cell mRNAs on single-cell virus yield (related to Figure 3). (**A**) Single non-infected MDCK cells were investigated for intracellular 18S rRNA via real-time RT-qPCR. The ribosome quantity was estimated as described in Figure 3A. The pooled data of two independent experiments are shown. n_S indicates the number of single-cell measurements. (**B**) Host cell and viral mRNA content of infected ("INF") and non-infected ("non-INF") cell population samples. For "INF", MDCK cells were infected with PR8 virus at an MOI of 10 and incubated until 12 hpi. Intracellular host cell and viral mRNA were analyzed by bulk RNA-seq. The pooled data of two independent experiments are shown. ***, $p<0.0005$ by Wilcoxon rank sum test. (**C** and **D**) Correlation between the single-cell virus titer (in PFU) and the host cell mRNA level (**C**) or the viral mRNA content (**D**). Single PR8-infected MDCK cells (MOI=10) were incubated until 12 hpi (as shown in Figure 1). Afterwards, virus yields were investigated using the plaque assay and intracellular host cell and viral mRNA were investigated via scRNA-seq. Cells were classified into low and high virus yields, as shown in Figure 3B. Spearman correlation coefficient is indicated. The pooled data of multiple independent experiments are shown (n=4).
